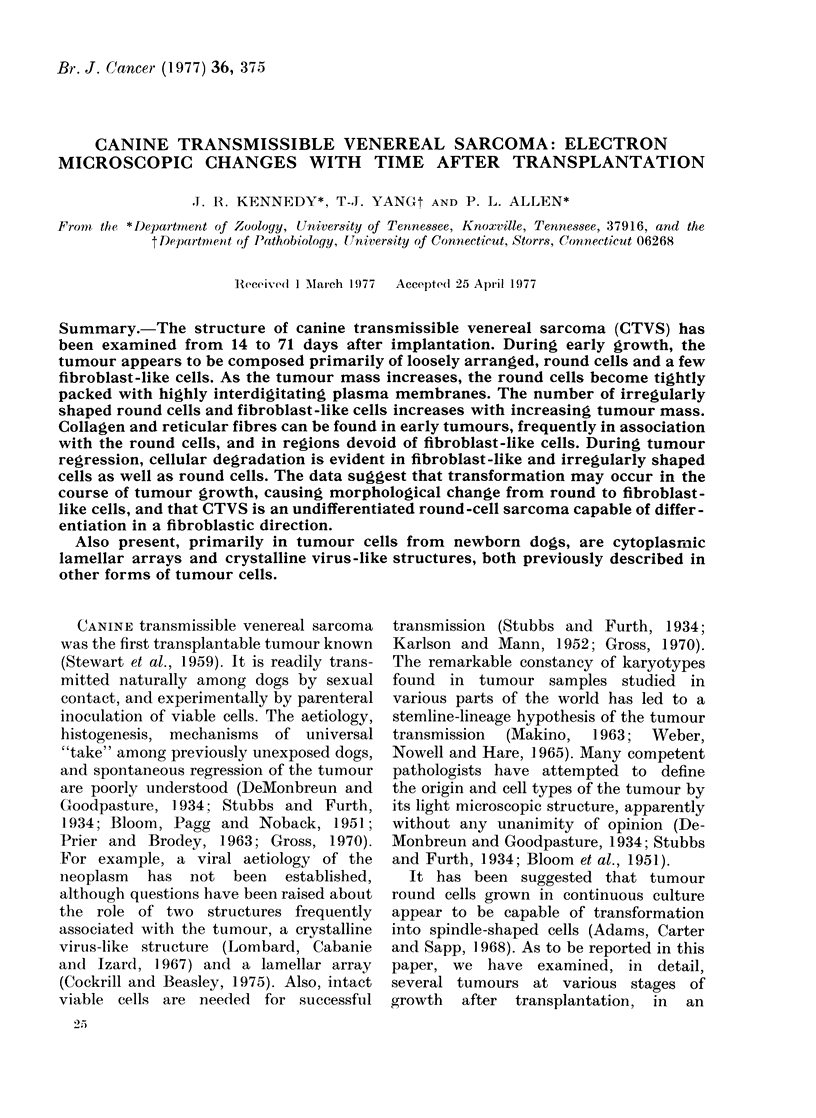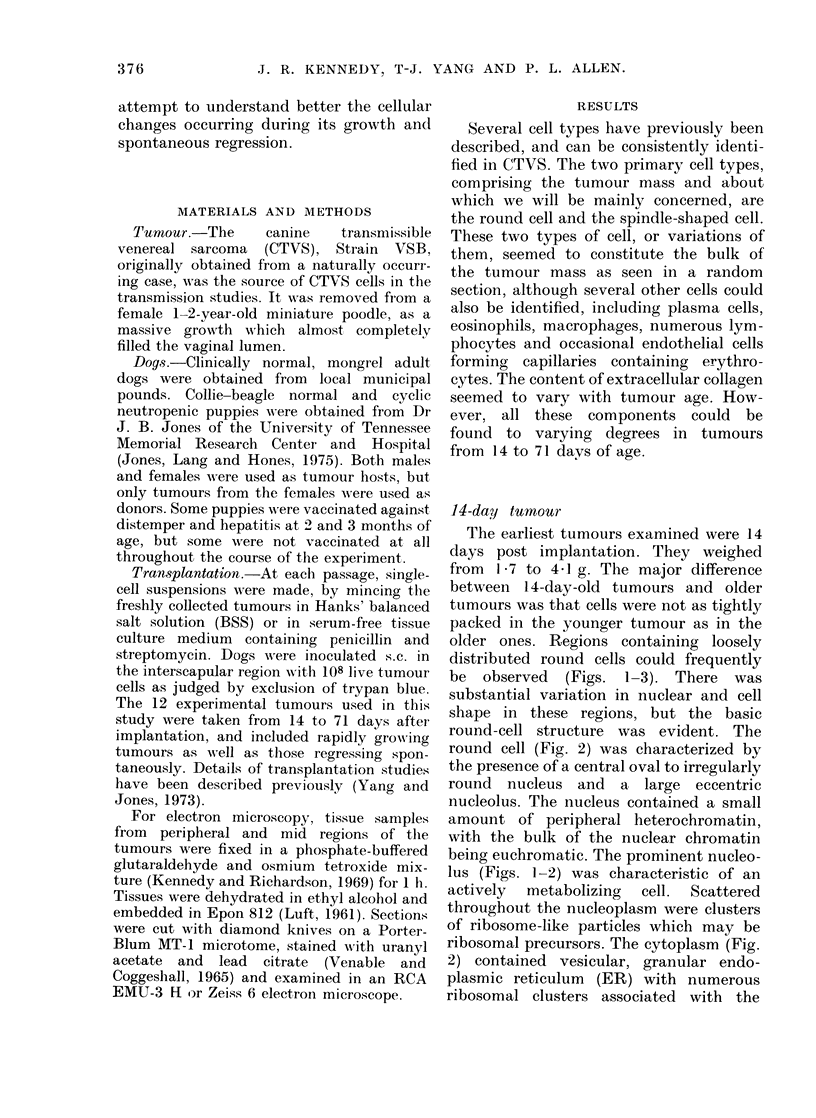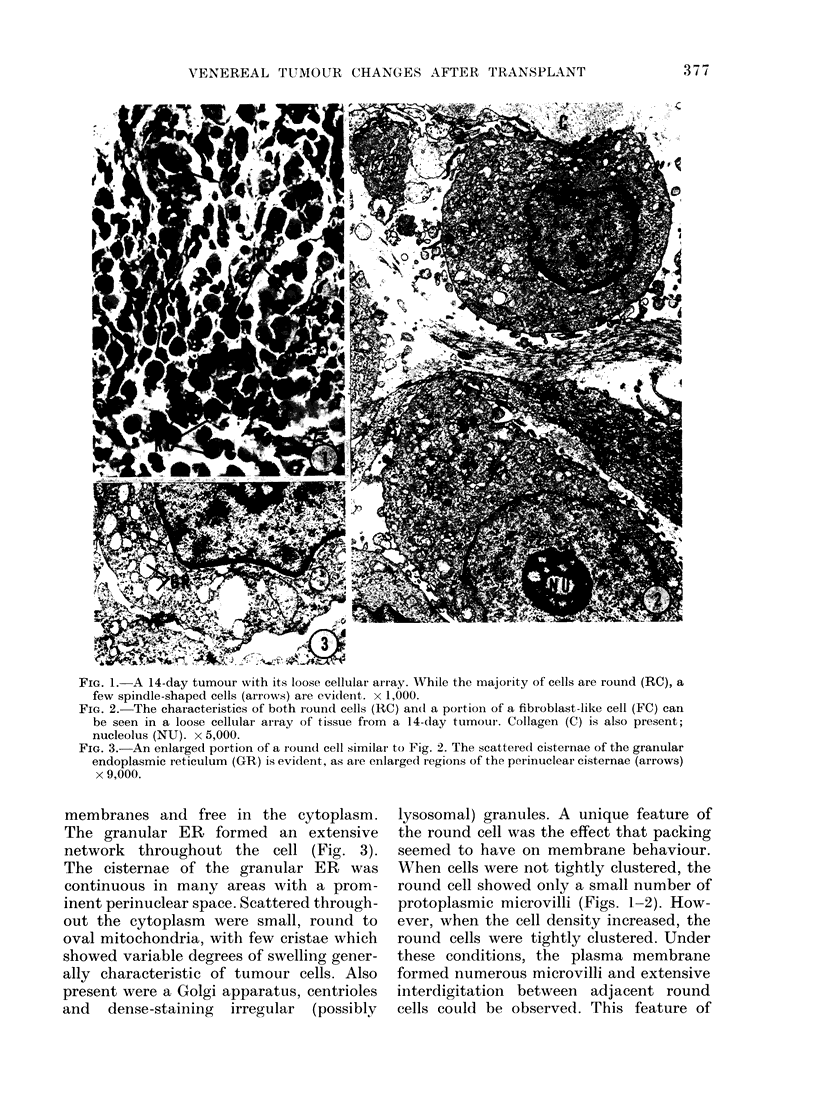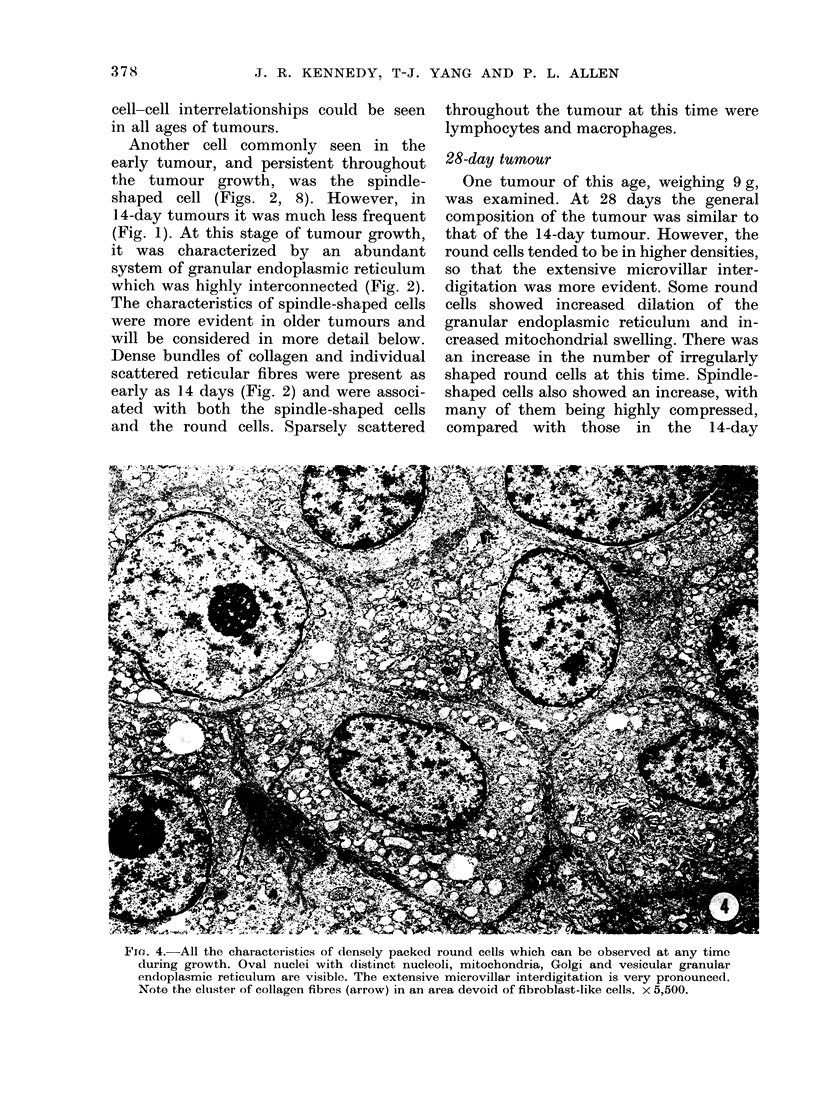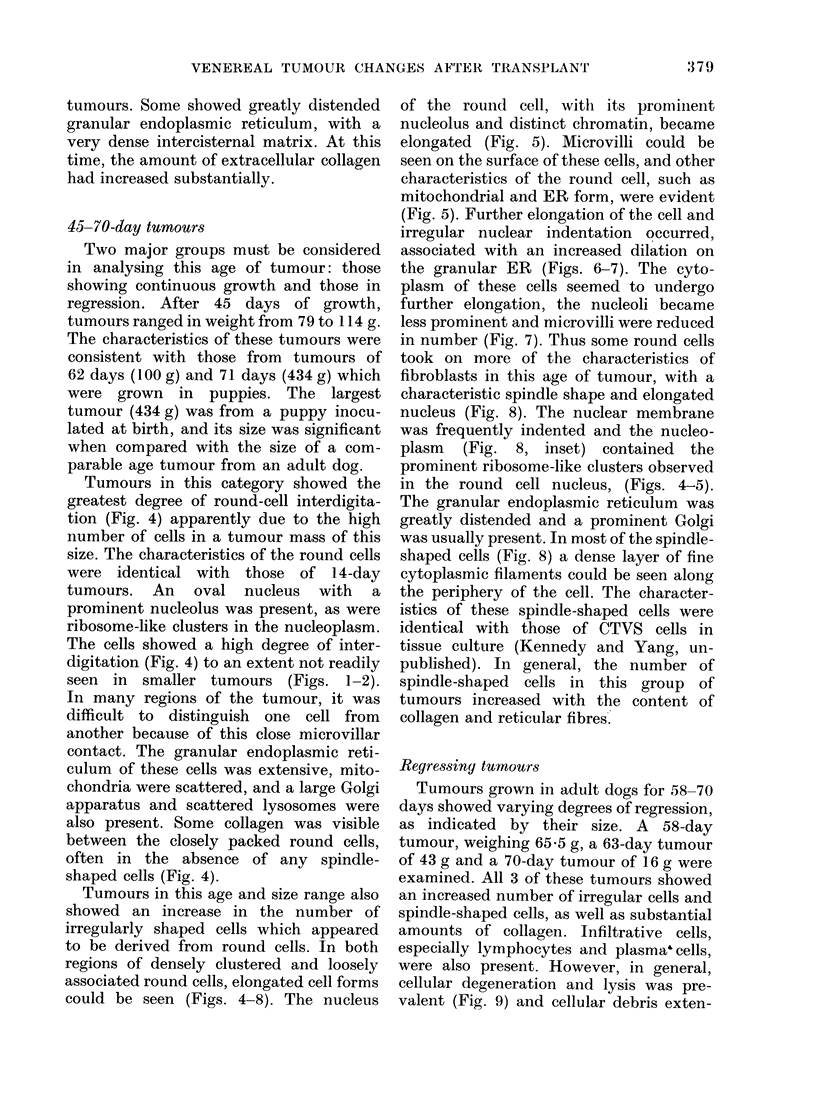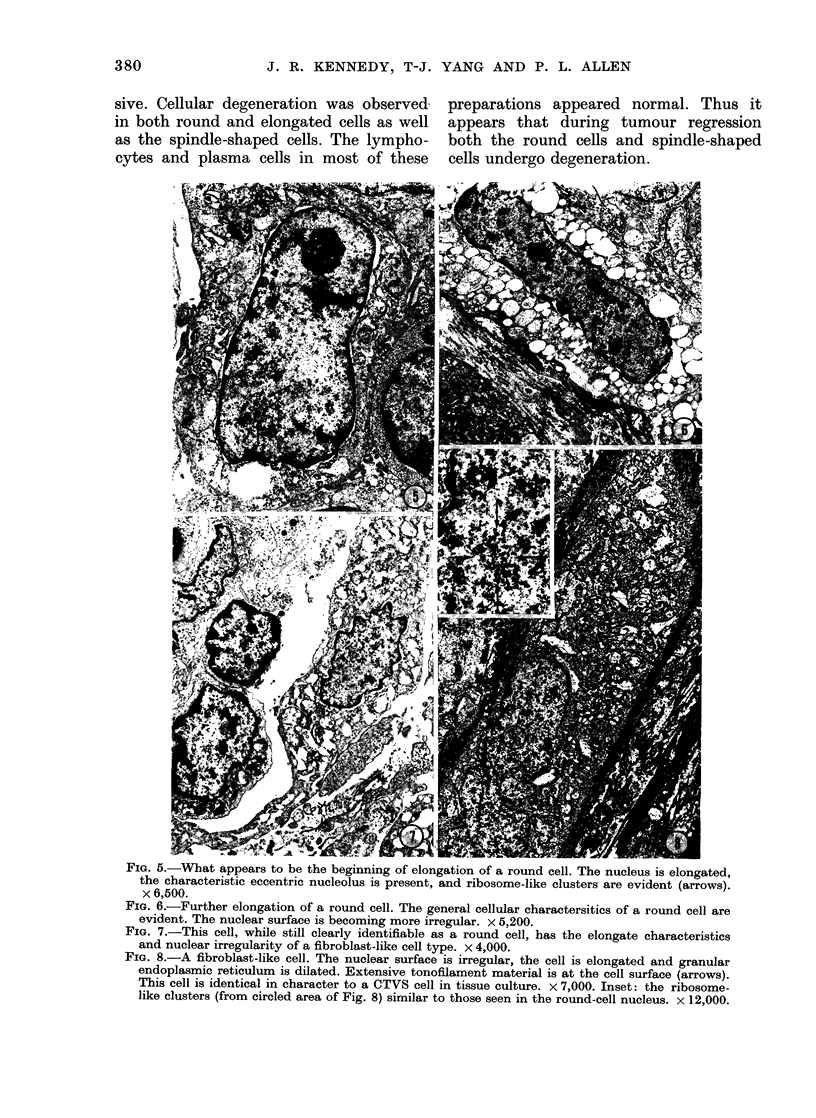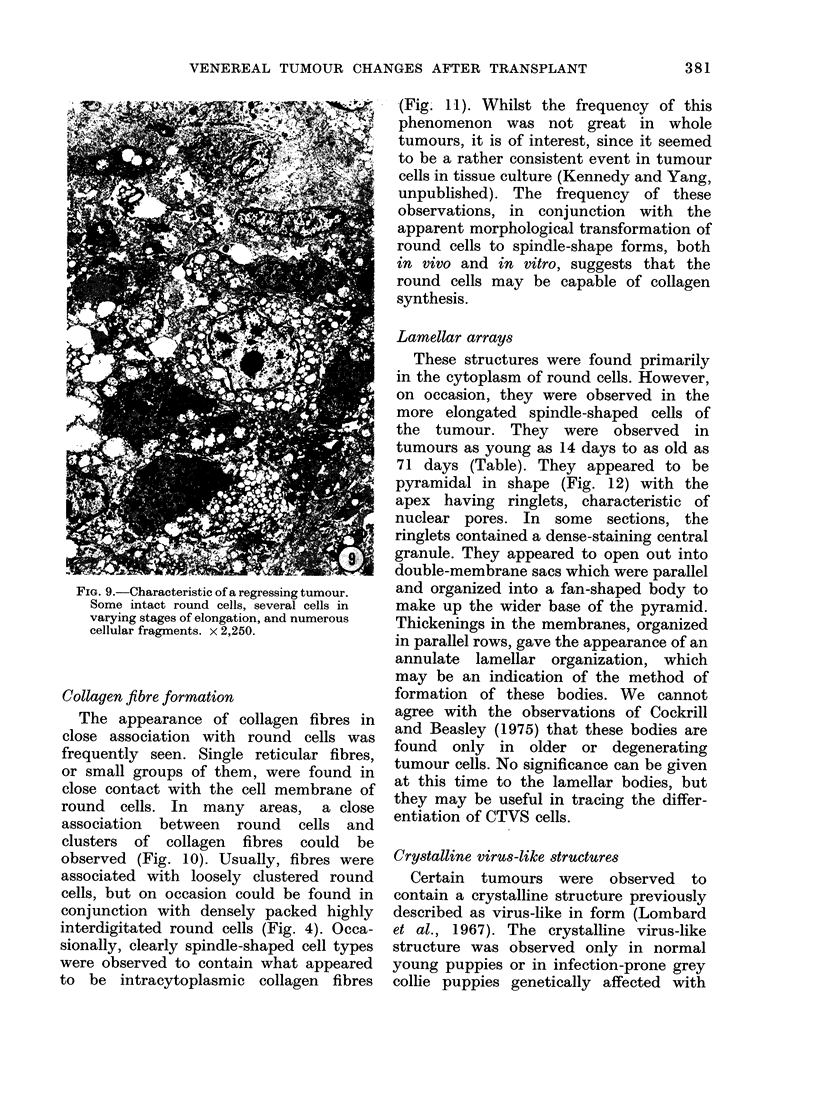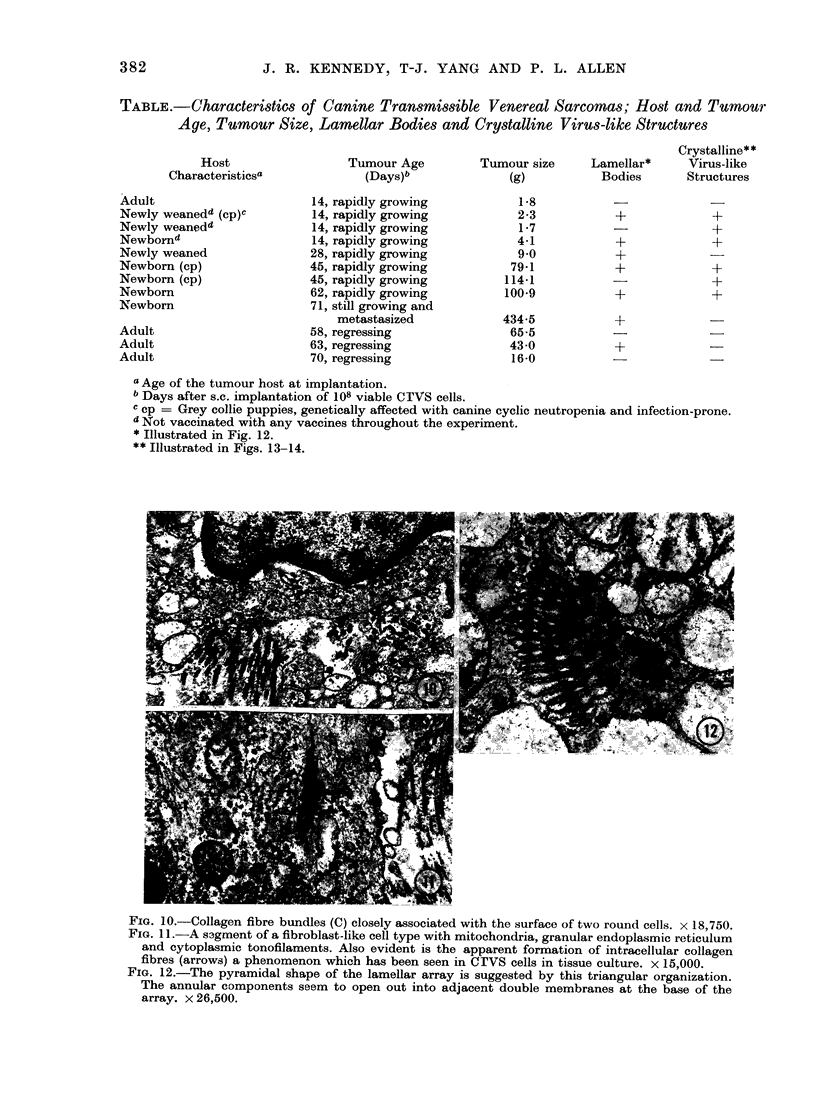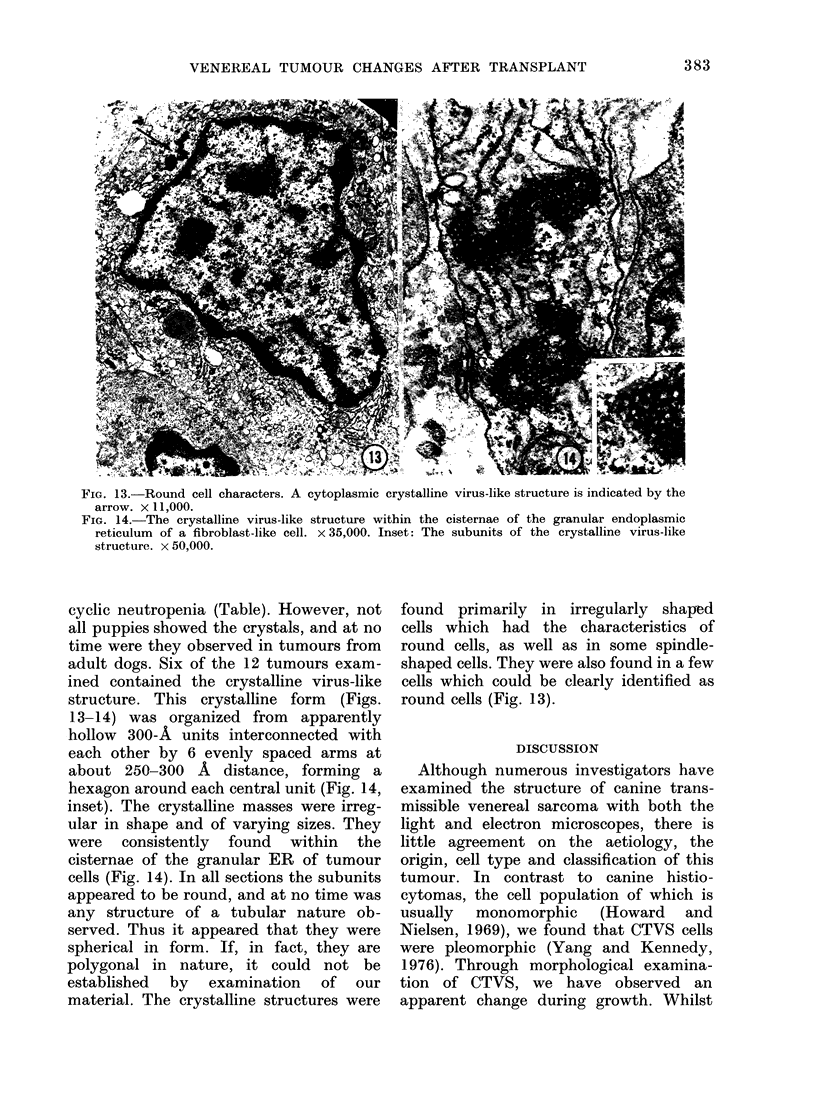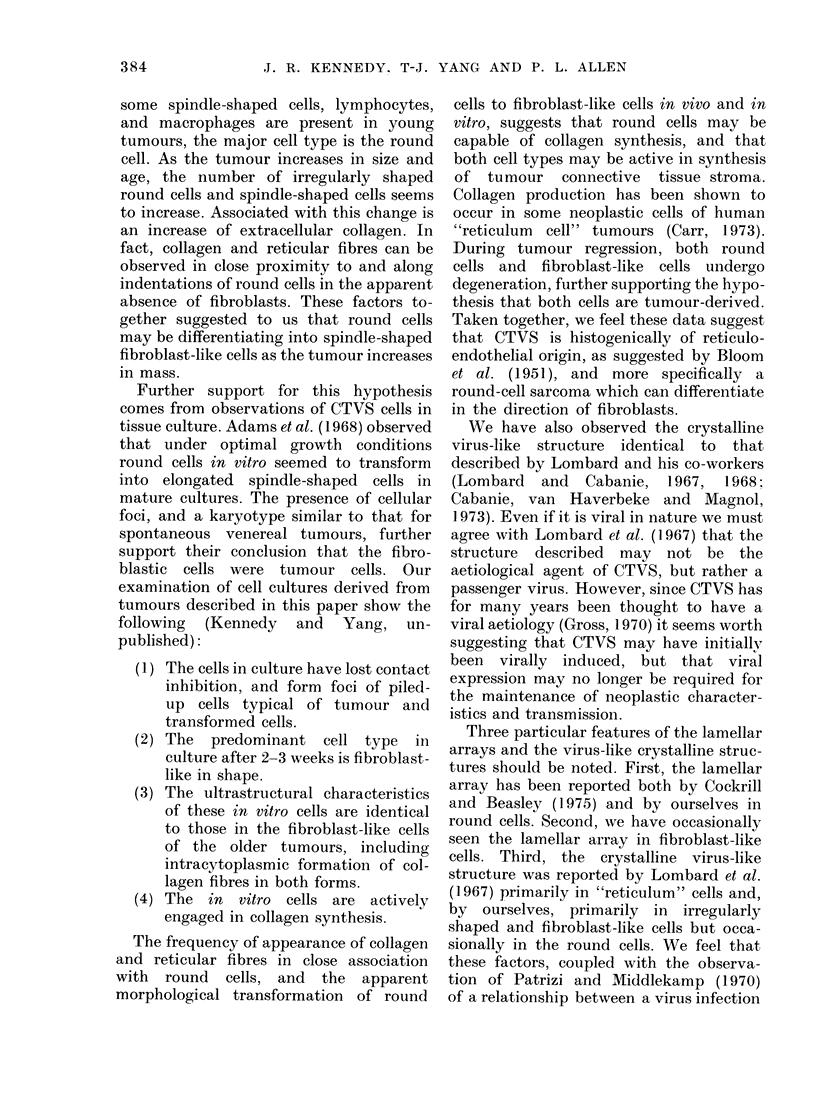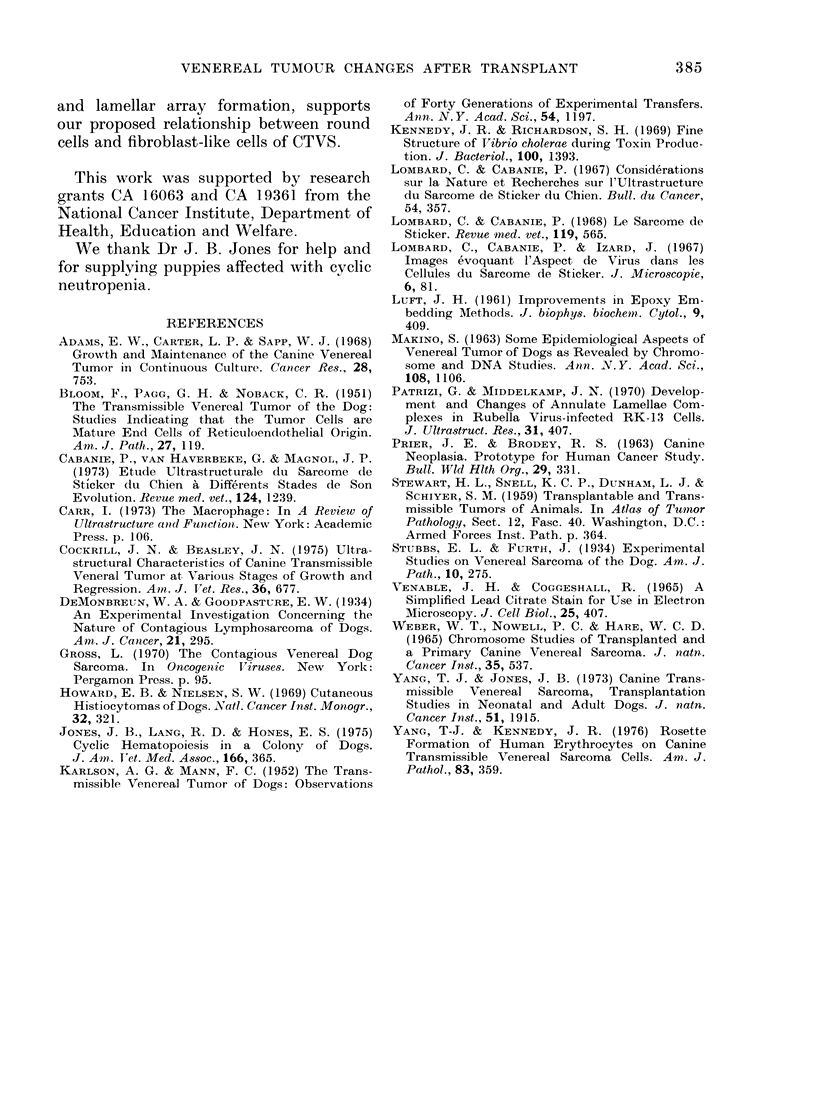# Canine transmissible venereal sarcoma: electron microscopic changes with time after transplantation.

**DOI:** 10.1038/bjc.1977.203

**Published:** 1977-09

**Authors:** J. R. Kennedy, T. J. Yang, P. L. Allen

## Abstract

**Images:**


*Br. J. Cancer* (1977) **36**, 375

### CANINE TRANSMISSIBLE VENEREAL SARCOMA: ELECTRON MICROSCOPIC CHANGES WITH TIME AFTER TRANSPLANTATION

J. R. KENNEDY*, T-J. YANG† AND P. L. ALLEN*

*From the *Department of Zoology, University of Tennessee, Knoxville, Tennessee,* 37916, *and the †Department of Pathobiology, University of Connecticut, Storrs, Connecticut* 06268

Received 1 March 1977 Accepted 25 April 1977

**Summary.—The structure of canine transmissible venereal sarcoma (CTVS) has been examined from 14 to 71 days after implantation. During early growth, the tumour appears to be composed primarily of loosely arranged, round cells and a few fibroblast-like cells. As the tumour mass increases, the round cells become tightly packed with highly interdigitating plasma membranes. The number of irregularly shaped round cells and fibroblast-like cells increases with increasing tumour mass. Collagen and reticular fibres can be found in early tumours, frequently in association with the round cells, and in regions devoid of fibroblast-like cells. During tumour regression, cellular degradation is evident in fibroblast-like and irregularly shaped cells as well as round cells. The data suggest that transformation may occur in the course of tumour growth, causing morphological change from round to fibroblast-like cells, and that CTVS is an undifferentiated round-cell sarcoma capable of differentiation in a fibroblastic direction.**

**Also present, primarily in tumour cells from newborn dogs, are cytoplasmic lamellar arrays and crystalline virus-like structures, both previously described in other forms of tumour cells.**

CANINE transmissible venereal sarcoma was the first transplantable tumour known (Stewart *et al.*, 1959). It is readily transmitted naturally among dogs by sexual contact, and experimentally by parenteral inoculation of viable cells. The aetiology, histogenesis, mechanisms of universal "take" among previously unexposed dogs, and spontaneous regression of the tumour are poorly understood (DeMonbreun and Goodpasture, 1934; Stubbs and Furth, 1934; Bloom, Pagg and Noback, 1951; Prier and Brodey, 1963; Gross, 1970). For example, a viral aetiology of the neoplasm has not been established, although questions have been raised about the role of two structures frequently associated with the tumour, a crystalline virus-like structure (Lombard, Cabanie and Izard, 1967) and a lamellar array (Cockrill and Beasley, 1975). Also, intact viable cells are needed for successful transmission (Stubbs and Furth, 1934; Karlson and Mann, 1952; Gross, 1970). The remarkable constancy of karyotypes found in tumour samples studied in various parts of the world has led to a stemline-lineage hypothesis of the tumour transmission (Makino, 1963; Weber, Nowell and Hare, 1965). Many competent pathologists have attempted to define the origin and cell types of the tumour by its light microscopic structure, apparently without any unanimity of opinion (DeMonbreun and Goodpasture, 1934; Stubbs and Furth, 1934; Bloom *et al.*, 1951).

It has been suggested that tumour round cells grown in continuous culture appear to be capable of transformation into spindle-shaped cells (Adams, Carter and Sapp, 1968). As to be reported in this paper, we have examined, in detail, several tumours at various stages of growth after transplantation, in an attempt to understand better the cellular changes occurring during its growth and spontaneous regression.

### MATERIALS AND METHODS

*Tumour.*—The canine transmissible venereal sarcoma (CTVS), Strain VSB, originally obtained from a naturally occurring case, was the source of CTVS cells in the transmission studies. It was removed from a female 1–2-year-old miniature poodle, as a massive growth which almost completely filled the vaginal lumen.

*Dogs.*—Clinically normal, mongrel adult dogs were obtained from local municipal pounds. Collie–beagle normal and cyclic neutropenic puppies were obtained from Dr J. B. Jones of the University of Tennessee Memorial Research Center and Hospital (Jones, Lang and Hones, 1975). Both males and females were used as tumour hosts, but only tumours from the females were used as donors. Some puppies were vaccinated against distemper and hepatitis at 2 and 3 months of age, but some were not vaccinated at all throughout the course of the experiment.

*Transplantation.*—At each passage, single-cell suspensions were made, by mincing the freshly collected tumours in Hanks' balanced salt solution (BSS) or in serum-free tissue culture medium containing penicillin and streptomycin. Dogs were inoculated s.c. in the interscapular region with $10^8$ live tumour cells as judged by exclusion of trypan blue. The 12 experimental tumours used in this study were taken from 14 to 71 days after implantation, and included rapidly growing tumours as well as those regressing spontaneously. Details of transplantation studies have been described previously (Yang and Jones, 1973).

For electron microscopy, tissue samples from peripheral and mid regions of the tumours were fixed in a phosphate-buffered glutaraldehyde and osmium tetroxide mixture (Kennedy and Richardson, 1969) for 1 h. Tissues were dehydrated in ethyl alcohol and embedded in Epon 812 (Luft, 1961). Sections were cut with diamond knives on a Porter-Blum MT-1 microtome, stained with uranyl acetate and lead citrate (Venable and Coggeshall, 1965) and examined in an RCA EMU-3 H or Zeiss 6 electron microscope.

### RESULTS

Several cell types have previously been described, and can be consistently identified in CTVS. The two primary cell types, comprising the tumour mass and about which we will be mainly concerned, are the round cell and the spindle-shaped cell. These two types of cell, or variations of them, seemed to constitute the bulk of the tumour mass as seen in a random section, although several other cells could also be identified, including plasma cells, eosinophils, macrophages, numerous lymphocytes and occasional endothelial cells forming capillaries containing erythrocytes. The content of extracellular collagen seemed to vary with tumour age. However, all these components could be found to varying degrees in tumours from 14 to 71 days of age.

### *14-day tumour*

The earliest tumours examined were 14 days post implantation. They weighed from 1·7 to 4·1 g. The major difference between 14-day-old tumours and older tumours was that cells were not as tightly packed in the younger tumour as in the older ones. Regions containing loosely distributed round cells could frequently be observed (Figs. 1–3). There was substantial variation in nuclear and cell shape in these regions, but the basic round-cell structure was evident. The round cell (Fig. 2) was characterized by the presence of a central oval to irregularly round nucleus and a large eccentric nucleolus. The nucleus contained a small amount of peripheral heterochromatin, with the bulk of the nuclear chromatin being euchromatic. The prominent nucleolus (Figs. 1–2) was characteristic of an actively metabolizing cell. Scattered throughout the nucleoplasm were clusters of ribosome-like particles which may be ribosomal precursors. The cytoplasm (Fig. 2) contained vesicular, granular endoplasmic reticulum (ER) with numerous ribosomal clusters associated with the

FIG. 1.—A 14-day tumour with its loose cellular array. While the majority of cells are round (RC), a few spindle-shaped cells (arrows) are evident. ×1,000.

FIG. 2.—The characteristics of both round cells (RC) and a portion of a fibroblast-like cell (FC) can be seen in a loose cellular array of tissue from a 14-day tumour. Collagen (C) is also present; nucleolus (NU). ×5,000.

FIG. 3.—An enlarged portion of a round cell similar to Fig. 2. The scattered cisternae of the granular endoplasmic reticulum (GR) is evident, as are enlarged regions of the perinuclear cisternae (arrows) ×9,000.

membranes and free in the cytoplasm. The granular ER formed an extensive network throughout the cell (Fig. 3). The cisternae of the granular ER was continuous in many areas with a prominent perinuclear space. Scattered throughout the cytoplasm were small, round to oval mitochondria, with few cristae which showed variable degrees of swelling generally characteristic of tumour cells. Also present were a Golgi apparatus, centrioles and dense-staining irregular (possibly lysosomal) granules. A unique feature of the round cell was the effect that packing seemed to have on membrane behaviour. When cells were not tightly clustered, the round cell showed only a small number of protoplasmic microvilli (Figs. 1–2). However, when the cell density increased, the round cells were tightly clustered. Under these conditions, the plasma membrane formed numerous microvilli and extensive interdigitation between adjacent round cells could be observed. This feature of cell–cell interrelationships could be seen in all ages of tumours.

Another cell commonly seen in the early tumour, and persistent throughout the tumour growth, was the spindle-shaped cell (Figs. 2, 8). However, in 14-day tumours it was much less frequent (Fig. 1). At this stage of tumour growth, it was characterized by an abundant system of granular endoplasmic reticulum which was highly interconnected (Fig. 2). The characteristics of spindle-shaped cells were more evident in older tumours and will be considered in more detail below. Dense bundles of collagen and individual scattered reticular fibres were present as early as 14 days (Fig. 2) and were associated with both the spindle-shaped cells and the round cells. Sparsely scattered throughout the tumour at this time were lymphocytes and macrophages.

*28-day tumour*

One tumour of this age, weighing 9 g, was examined. At 28 days the general composition of the tumour was similar to that of the 14-day tumour. However, the round cells tended to be in higher densities, so that the extensive microvillar interdigitation was more evident. Some round cells showed increased dilation of the granular endoplasmic reticulum and increased mitochondrial swelling. There was an increase in the number of irregularly shaped round cells at this time. Spindle-shaped cells also showed an increase, with many of them being highly compressed, compared with those in the 14-day

Fig. 4.—All the characteristics of densely packed round cells which can be observed at any time during growth. Oval nuclei with distinct nucleoli, mitochondria, Golgi and vesicular granular endoplasmic reticulum are visible. The extensive microvillar interdigitation is very pronounced. Note the cluster of collagen fibres (arrow) in an area devoid of fibroblast-like cells. $\times$ 5,500.

tumours. Some showed greatly distended granular endoplasmic reticulum, with a very dense intercisternal matrix. At this time, the amount of extracellular collagen had increased substantially.

### *45–70-day tumours*

Two major groups must be considered in analysing this age of tumour: those showing continuous growth and those in regression. After 45 days of growth, tumours ranged in weight from 79 to 114 g. The characteristics of these tumours were consistent with those from tumours of 62 days (100 g) and 71 days (434 g) which were grown in puppies. The largest tumour (434 g) was from a puppy inoculated at birth, and its size was significant when compared with the size of a comparable age tumour from an adult dog.

Tumours in this category showed the greatest degree of round-cell interdigitation (Fig. 4) apparently due to the high number of cells in a tumour mass of this size. The characteristics of the round cells were identical with those of 14-day tumours. An oval nucleus with a prominent nucleolus was present, as were ribosome-like clusters in the nucleoplasm. The cells showed a high degree of interdigitation (Fig. 4) to an extent not readily seen in smaller tumours (Figs. 1–2). In many regions of the tumour, it was difficult to distinguish one cell from another because of this close microvillar contact. The granular endoplasmic reticulum of these cells was extensive, mitochondria were scattered, and a large Golgi apparatus and scattered lysosomes were also present. Some collagen was visible between the closely packed round cells, often in the absence of any spindle-shaped cells (Fig. 4).

Tumours in this age and size range also showed an increase in the number of irregularly shaped cells which appeared to be derived from round cells. In both regions of densely clustered and loosely associated round cells, elongated cell forms could be seen (Figs. 4–8). The nucleus of the round cell, with its prominent nucleolus and distinct chromatin, became elongated (Fig. 5). Microvilli could be seen on the surface of these cells, and other characteristics of the round cell, such as mitochondrial and ER form, were evident (Fig. 5). Further elongation of the cell and irregular nuclear indentation occurred, associated with an increased dilation on the granular ER (Figs. 6–7). The cytoplasm of these cells seemed to undergo further elongation, the nucleoli became less prominent and microvilli were reduced in number (Fig. 7). Thus some round cells took on more of the characteristics of fibroblasts in this age of tumour, with a characteristic spindle shape and elongated nucleus (Fig. 8). The nuclear membrane was frequently indented and the nucleoplasm (Fig. 8, inset) contained the prominent ribosome-like clusters observed in the round cell nucleus, (Figs. 4–5). The granular endoplasmic reticulum was greatly distended and a prominent Golgi was usually present. In most of the spindle-shaped cells (Fig. 8) a dense layer of fine cytoplasmic filaments could be seen along the periphery of the cell. The characteristics of these spindle-shaped cells were identical with those of CTVS cells in tissue culture (Kennedy and Yang, unpublished). In general, the number of spindle-shaped cells in this group of tumours increased with the content of collagen and reticular fibres.

### *Regressing tumours*

Tumours grown in adult dogs for 58–70 days showed varying degrees of regression, as indicated by their size. A 58-day tumour, weighing 65·5 g, a 63-day tumour of 43 g and a 70-day tumour of 16 g were examined. All 3 of these tumours showed an increased number of irregular cells and spindle-shaped cells, as well as substantial amounts of collagen. Infiltrative cells, especially lymphocytes and plasma cells, were also present. However, in general, cellular degeneration and lysis was prevalent (Fig. 9) and cellular debris extensive. Cellular degeneration was observed in both round and elongated cells as well as the spindle-shaped cells. The lymphocytes and plasma cells in most of these preparations appeared normal. Thus it appears that during tumour regression both the round cells and spindle-shaped cells undergo degeneration.

FIG. 5.—What appears to be the beginning of elongation of a round cell. The nucleus is elongated, the characteristic eccentric nucleolus is present, and ribosome-like clusters are evident (arrows). ×6,500.

FIG. 6.—Further elongation of a round cell. The general cellular charactersitics of a round cell are evident. The nuclear surface is becoming more irregular. ×5,200.

FIG. 7.—This cell, while still clearly identifiable as a round cell, has the elongate characteristics and nuclear irregularity of a fibroblast-like cell type. ×4,000.

FIG. 8.—A fibroblast-like cell. The nuclear surface is irregular, the cell is elongated and granular endoplasmic reticulum is dilated. Extensive tonofilament material is at the cell surface (arrows). This cell is identical in character to a CTVS cell in tissue culture. ×7,000. Inset: the ribosome-like clusters (from circled area of Fig. 8) similar to those seen in the round-cell nucleus. ×12,000.

FIG. 9.—Characteristic of a regressing tumour. Some intact round cells, several cells in varying stages of elongation, and numerous cellular fragments. ×2,250.

*Collagen fibre formation*

The appearance of collagen fibres in close association with round cells was frequently seen. Single reticular fibres, or small groups of them, were found in close contact with the cell membrane of round cells. In many areas, a close association between round cells and clusters of collagen fibres could be observed (Fig. 10). Usually, fibres were associated with loosely clustered round cells, but on occasion could be found in conjunction with densely packed highly interdigitated round cells (Fig. 4). Occasionally, clearly spindle-shaped cell types were observed to contain what appeared to be intracytoplasmic collagen fibres (Fig. 11). Whilst the frequency of this phenomenon was not great in whole tumours, it is of interest, since it seemed to be a rather consistent event in tumour cells in tissue culture (Kennedy and Yang, unpublished). The frequency of these observations, in conjunction with the apparent morphological transformation of round cells to spindle-shape forms, both *in vivo* and *in vitro*, suggests that the round cells may be capable of collagen synthesis.

*Lamellar arrays*

These structures were found primarily in the cytoplasm of round cells. However, on occasion, they were observed in the more elongated spindle-shaped cells of the tumour. They were observed in tumours as young as 14 days to as old as 71 days (Table). They appeared to be pyramidal in shape (Fig. 12) with the apex having ringlets, characteristic of nuclear pores. In some sections, the ringlets contained a dense-staining central granule. They appeared to open out into double-membrane sacs which were parallel and organized into a fan-shaped body to make up the wider base of the pyramid. Thickenings in the membranes, organized in parallel rows, gave the appearance of an annulate lamellar organization, which may be an indication of the method of formation of these bodies. We cannot agree with the observations of Cockrill and Beasley (1975) that these bodies are found only in older or degenerating tumour cells. No significance can be given at this time to the lamellar bodies, but they may be useful in tracing the differentiation of CTVS cells.

*Crystalline virus-like structures*

Certain tumours were observed to contain a crystalline structure previously described as virus-like in form (Lombard *et al.*, 1967). The crystalline virus-like structure was observed only in normal young puppies or in infection-prone grey collie puppies genetically affected with

TABLE.—*Characteristics of Canine Transmissible Venereal Sarcomas; Host and Tumour Age, Tumour Size, Lamellar Bodies and Crystalline Virus-like Structures*

| Host Characteristics[a] | Tumour Age (Days)[b] | Tumour size (g) | Lamellar* Bodies | Crystalline** Virus-like Structures |
|---|---|---|---|---|
| Adult | 14, rapidly growing | 1·8 | — | — |
| Newly weaned[d] (cp)[c] | 14, rapidly growing | 2·3 | + | + |
| Newly weaned[d] | 14, rapidly growing | 1·7 | — | + |
| Newborn[d] | 14, rapidly growing | 4·1 | + | + |
| Newly weaned | 28, rapidly growing | 9·0 | + | — |
| Newborn (cp) | 45, rapidly growing | 79·1 | + | + |
| Newborn (cp) | 45, rapidly growing | 114·1 | — | + |
| Newborn | 62, rapidly growing | 100·9 | + | + |
| Newborn | 71, still growing and metastasized | 434·5 | + | — |
| Adult | 58, regressing | 65·5 | — | — |
| Adult | 63, regressing | 43·0 | + | — |
| Adult | 70, regressing | 16·0 | — | — |

[a] Age of the tumour host at implantation.
[b] Days after s.c. implantation of $10^8$ viable CTVS cells.
[c] cp = Grey collie puppies, genetically affected with canine cyclic neutropenia and infection-prone.
[d] Not vaccinated with any vaccines throughout the experiment.
* Illustrated in Fig. 12.
** Illustrated in Figs. 13–14.

FIG. 10.—Collagen fibre bundles (C) closely associated with the surface of two round cells. ×18,750.

FIG. 11.—A segment of a fibroblast-like cell type with mitochondria, granular endoplasmic reticulum and cytoplasmic tonofilaments. Also evident is the apparent formation of intracellular collagen fibres (arrows) a phenomenon which has been seen in CTVS cells in tissue culture. ×15,000.

FIG. 12.—The pyramidal shape of the lamellar array is suggested by this triangular organization. The annular components seem to open out into adjacent double membranes at the base of the array. ×26,500.

FIG. 13.—Round cell characters. A cytoplasmic crystalline virus-like structure is indicated by the arrow. × 11,000.

FIG. 14.—The crystalline virus-like structure within the cisternae of the granular endoplasmic reticulum of a fibroblast-like cell. × 35,000. Inset: The subunits of the crystalline virus-like structure. × 50,000.

cyclic neutropenia (Table). However, not all puppies showed the crystals, and at no time were they observed in tumours from adult dogs. Six of the 12 tumours examined contained the crystalline virus-like structure. This crystalline form (Figs. 13–14) was organized from apparently hollow 300-Å units interconnected with each other by 6 evenly spaced arms at about 250–300 Å distance, forming a hexagon around each central unit (Fig. 14, inset). The crystalline masses were irregular in shape and of varying sizes. They were consistently found within the cisternae of the granular ER of tumour cells (Fig. 14). In all sections the subunits appeared to be round, and at no time was any structure of a tubular nature observed. Thus it appeared that they were spherical in form. If, in fact, they are polygonal in nature, it could not be established by examination of our material. The crystalline structures were found primarily in irregularly shaped cells which had the characteristics of round cells, as well as in some spindle-shaped cells. They were also found in a few cells which could be clearly identified as round cells (Fig. 13).

### DISCUSSION

Although numerous investigators have examined the structure of canine transmissible venereal sarcoma with both the light and electron microscopes, there is little agreement on the aetiology, the origin, cell type and classification of this tumour. In contrast to canine histiocytomas, the cell population of which is usually monomorphic (Howard and Nielsen, 1969), we found that CTVS cells were pleomorphic (Yang and Kennedy, 1976). Through morphological examination of CTVS, we have observed an apparent change during growth. Whilst some spindle-shaped cells, lymphocytes, and macrophages are present in young tumours, the major cell type is the round cell. As the tumour increases in size and age, the number of irregularly shaped round cells and spindle-shaped cells seems to increase. Associated with this change is an increase of extracellular collagen. In fact, collagen and reticular fibres can be observed in close proximity to and along indentations of round cells in the apparent absence of fibroblasts. These factors together suggested to us that round cells may be differentiating into spindle-shaped fibroblast-like cells as the tumour increases in mass.

Further support for this hypothesis comes from observations of CTVS cells in tissue culture. Adams *et al.* (1968) observed that under optimal growth conditions round cells *in vitro* seemed to transform into elongated spindle-shaped cells in mature cultures. The presence of cellular foci, and a karyotype similar to that for spontaneous venereal tumours, further support their conclusion that the fibroblastic cells were tumour cells. Our examination of cell cultures derived from tumours described in this paper show the following (Kennedy and Yang, unpublished):

(1) The cells in culture have lost contact inhibition, and form foci of piled-up cells typical of tumour and transformed cells.
(2) The predominant cell type in culture after 2–3 weeks is fibroblast-like in shape.
(3) The ultrastructural characteristics of these *in vitro* cells are identical to those in the fibroblast-like cells of the older tumours, including intracytoplasmic formation of collagen fibres in both forms.
(4) The *in vitro* cells are actively engaged in collagen synthesis.

The frequency of appearance of collagen and reticular fibres in close association with round cells, and the apparent morphological transformation of round cells to fibroblast-like cells *in vivo* and *in vitro*, suggests that round cells may be capable of collagen synthesis, and that both cell types may be active in synthesis of tumour connective tissue stroma. Collagen production has been shown to occur in some neoplastic cells of human "reticulum cell" tumours (Carr, 1973). During tumour regression, both round cells and fibroblast-like cells undergo degeneration, further supporting the hypothesis that both cells are tumour-derived. Taken together, we feel these data suggest that CTVS is histogenically of reticulo-endothelial origin, as suggested by Bloom *et al.* (1951), and more specifically a round-cell sarcoma which can differentiate in the direction of fibroblasts.

We have also observed the crystalline virus-like structure identical to that described by Lombard and his co-workers (Lombard and Cabanie, 1967, 1968; Cabanie, van Haverbeke and Magnol, 1973). Even if it is viral in nature we must agree with Lombard *et al.* (1967) that the structure described may not be the aetiological agent of CTVS, but rather a passenger virus. However, since CTVS has for many years been thought to have a viral aetiology (Gross, 1970) it seems worth suggesting that CTVS may have initially been virally induced, but that viral expression may no longer be required for the maintenance of neoplastic characteristics and transmission.

Three particular features of the lamellar arrays and the virus-like crystalline structures should be noted. First, the lamellar array has been reported both by Cockrill and Beasley (1975) and by ourselves in round cells. Second, we have occasionally seen the lamellar array in fibroblast-like cells. Third, the crystalline virus-like structure was reported by Lombard *et al.* (1967) primarily in "reticulum" cells and, by ourselves, primarily in irregularly shaped and fibroblast-like cells but occasionally in the round cells. We feel that these factors, coupled with the observation of Patrizi and Middlekamp (1970) of a relationship between a virus infection and lamellar array formation, supports our proposed relationship between round cells and fibroblast-like cells of CTVS.

This work was supported by research grants CA 16063 and CA 19361 from the National Cancer Institute, Department of Health, Education and Welfare.

We thank Dr J. B. Jones for help and for supplying puppies affected with cyclic neutropenia.

### REFERENCES

ADAMS, E. W., CARTER, L. P. & SAPP, W. J. (1968) Growth and Maintenance of the Canine Venereal Tumor in Continuous Culture. *Cancer Res.*, **28**, 753.

BLOOM, F., PAGG, G. H. & NOBACK, C. R. (1951) The Transmissible Venereal Tumor of the Dog: Studies Indicating that the Tumor Cells are Mature End Cells of Reticuloendothelial Origin. *Am. J. Path.*, **27**, 119.

CABANIE, P., VAN HAVERBEKE, G. & MAGNOL, J. P. (1973) Etude Ultrastructurale du Sarcome de Sticker du Chien à Différents Stades de Son Evolution. *Revue med. vet.*, **124**, 1239.

CARR, I. (1973) The Macrophage: In *A Review of Ultrastructure and Function*. New York: Academic Press. p. 106.

COCKRILL, J. N. & BEASLEY, J. N. (1975) Ultrastructural Characteristics of Canine Transmissible Veneral Tumor at Various Stages of Growth and Regression. *Am. J. Vet. Res.*, **36**, 677.

DEMONBREUN, W. A. & GOODPASTURE, E. W. (1934) An Experimental Investigation Concerning the Nature of Contagious Lymphosarcoma of Dogs. *Am. J. Cancer*, **21**, 295.

GROSS, L. (1970) The Contagious Venereal Dog Sarcoma. In *Oncogenic Viruses*. New York: Pergamon Press. p. 95.

HOWARD, E. B. & NIELSEN, S. W. (1969) Cutaneous Histiocytomas of Dogs. *Natl. Cancer Inst. Monogr.*, **32**, 321.

JONES, J. B., LANG, R. D. & HONES, E. S. (1975) Cyclic Hematopoiesis in a Colony of Dogs. *J. Am. Vet. Med. Assoc.*, **166**, 365.

KARLSON, A. G. & MANN, F. C. (1952) The Transmissible Venereal Tumor of Dogs: Observations of Forty Generations of Experimental Transfers. *Ann. N.Y. Acad. Sci.*, **54**, 1197.

KENNEDY, J. R. & RICHARDSON, S. H. (1969) Fine Structure of *Vibrio cholerae* during Toxin Production. *J. Bacteriol.*, **100**, 1393.

LOMBARD, C. & CABANIE, P. (1967) Considérations sur la Nature et Recherches sur l'Ultrastructure du Sarcome de Sticker du Chien. *Bull. du Cancer*, 54, 357.

LOMBARD, C. & CABANIE, P. (1968) Le Sarcome de Sticker. *Revue med. vet.*, **119**, 565.

LOMBARD, C., CABANIE, P. & IZARD, J. (1967) Images évoquant l'Aspect de Virus dans les Cellules du Sarcome de Sticker. *J. Microscopie*, **6**, 81.

LUFT, J. H. (1961) Improvements in Epoxy Embedding Methods. *J. biophys. biochem. Cytol.*, **9**, 409.

MAKINO, S. (1963) Some Epidemiological Aspects of Venereal Tumor of Dogs as Revealed by Chromosome and DNA Studies. *Ann. N.Y. Acad. Sci.*, **108**, 1106.

PATRIZI, G. & MIDDELKAMP, J. N. (1970) Development and Changes of Annulate Lamellae Complexes in Rubella Virus-infected RK-13 Cells. *J. Ultrastruct. Res.*, **31**, 407.

PRIER, J. E. & BRODEY, R. S. (1963) Canine Neoplasia. Prototype for Human Cancer Study. *Bull. Wld Hlth Org.*, **29**, 331.

STEWART, H. L., SNELL, K. C. P., DUNHAM, L. J. & SCHIYER, S. M. (1959) Transplantable and Transmissible Tumors of Animals. In *Atlas of Tumor Pathology*, Sect. 12, Fasc. 40. Washington, D.C.: Armed Forces Inst. Path. p. 364.

STUBBS, E. L. & FURTH, J. (1934) Experimental Studies on Venereal Sarcoma of the Dog. *Am. J. Path.*, **10**, 275.

VENABLE, J. H. & COGGESHALL, R. (1965) A Simplified Lead Citrate Stain for Use in Electron Microscopy. *J. Cell Biol.*, **25**, 407.

WEBER, W. T., NOWELL, P. C. & HARE, W. C. D. (1965) Chromosome Studies of Transplanted and a Primary Canine Venereal Sarcoma. *J. natn. Cancer Inst.*, **35**, 537.

YANG, T. J. & JONES, J. B. (1973) Canine Transmissible Venereal Sarcoma, Transplantation Studies in Neonatal and Adult Dogs. *J. natn. Cancer Inst.*, **51**, 1915.

YANG, T-J. & KENNEDY, J. R. (1976) Rosette Formation of Human Erythrocytes on Canine Transmissible Venereal Sarcoma Cells. *Am. J. Pathol.*, **83**, 359.